# Feature selection algorithm based on optimized genetic algorithm and the application in high-dimensional data processing

**DOI:** 10.1371/journal.pone.0303088

**Published:** 2024-05-09

**Authors:** Guilian Feng

**Affiliations:** School of Physics and Electronic Information Engineering, Qinghai Minzu University, Xining, China; Air University, PAKISTAN

## Abstract

High-dimensional data is widely used in many fields, but selecting key features from it is challenging. Feature selection can reduce data dimensionality and weaken noise interference, thereby improving model efficiency and enhancing model interpretability. In order to improve the efficiency and accuracy of high-dimensional data processing, a feature selection method based on optimized genetic algorithm is proposed in this study. The algorithm simulates the process of natural selection, searches for possible subsets of feature, and finds the subsets of feature that optimizes the performance of the model. The results show that when the value of K is less than 4 or more than 8, the recognition rate is very low. After adaptive bias filtering, 724 features are filtered to 372, and the accuracy is improved from 0.9352 to 0.9815. From 714 features to 406 Gaussian codes, the accuracy is improved from 0.9625 to 0.9754. Among all tests, the colon has the highest average accuracy, followed by small round blue cell tumor(SRBCT), lymphoma, central nervous system(CNS) and ovaries. The green curve is the best, with stable performance and a time range of 0–300. While maintaining the efficiency, it can reach 4.48 as soon as possible. The feature selection method has practical significance for high-dimensional data processing, improves the efficiency and accuracy of data processing, and provides an effective new method for high-dimensional data processing.

## 1. Introduction

To address the challenges faced by high-dimensional data, appropriate data dimension reduction techniques can be adopted to reduce the data dimensions and preserve the most important information. These data often contain important information that helps with decision-making or prediction. However, due to the high dimensions, the efficiency and effectiveness of using all features for data analysis are not ideal. Sometimes, it can even reduce the predictive accuracy. Therefore, selecting important features to reduce data dimensions and improve model learning and prediction accuracy has become an important topic in high-dimensional data processing [1, 2]. Feature selection (FS) is an effective way to reduce data dimensions, but it is also an extremely complex task. Traditional FS methods, such as filtering methods and packaging method, are prone to low computational efficiency or difficulty in obtaining the global optimal solution [3, 4]. Genetic algorithm (GA) is a heuristic global optimization algorithm. It finds the optimal solution by simulating the biological evolution process in nature. It is widely used in many complex problems. The FS algorithm based on optimized GA and the application in high-dimensional data processing is proposed. The purpose is to propose a new FS algorithm by optimizing the traditional GA to improve the efficiency and accuracy of high-dimensional data processing [5, 6]. The contribution of the research lies in proposing a feature selection method for optimizing GA to address the challenges in high-dimensional data processing. Through in-depth research and improvement of the initialization process, crossover and mutation operations, and adaptive functions of GA, this method aims to improve search ability and convergence speed, and overcome dimensional issues. In addition, the effectiveness of this method in practical applications has been demonstrated through experimental verification on real high-dimensional datasets. The research is conducted in four parts. The first part is an overview of feature selection algorithms based on optimized GA and their applications in high-dimensional data processing. The second part is a feature selection algorithm based on optimized GA and its application in high-dimensional data processing. The third part is experimental verification for the second part. The fourth part is a summary of the research content and points out the shortcomings.

## 2. Related work

With the progress of social information technology, a large amount of data has been generated in daily life. Therefore, in rich data, many interfering data needs to be removed. GA is a global search algorithm proposed by John Holland in the University of Michigan in the United States. GA is used to solve function optimization problems in high-dimensional data, multi-objective problems, medical image feature extraction, machine learning, and neural network thresholds. However, GA has a slow search speed. It is also easy to fall into local optima during the search process [7]. The widespread popularity of technology and information technology has made the data volume grow exponentially. It brings advantages in data volume, but also has the disadvantage of diverse data features. It leads to more complex modeling in machine learning. Therefore, it is necessary to continuously update and optimize algorithms. FS is a common data pre-processing method in data mining. It aims to build better classifiers by listing salient features while reducing computational load. FS only maintains features with good capabilities. Describing inherent patterns within data based on important criteria can reduce the impact of dimensions [8].

Z Hong fang et al. proposed a weight composition feature relevance (WCFR) method based on weighted conditional information. Standard deviation (SD) is used to adjust the importance between correlation and redundancy. The SD is used to measure the relationship between features and feature sets. Mutual information is often used to measure the relationship between features and classes. The new WCFR method is an improvement on CFR. WCFR is more effective than other methods without increasing time complexity [9]. Z Ping et al. proposed the FS algorithm of uncertainty change rate by considering the uncertainty of class label reduction and class label residual under different feature conditions. As the selected features increases, the redundancy between feature subsets also increases. Moreover, the calculation form is relatively complex. There may be errors in measuring uncertainty [10]. L Jing et al. proposed a multi tag FS method for stream tagging. The method first selects specific features for each marker based on mutual information and discrimination index. Then, the labeled features produces the final feature subset, but such a feature subset cannot label accurate information features [11]. D K Rakesh et al. proposed a new FS method called class-label specific mutual information (CSMI). Mutual information is used to express the correlation between features and labels. This method maximizes the shared information between the selected feature and the target class label, while minimizing the shared information between all classes. However, the information processing and targeted optimization of FS algorithm are ignored [12]. Based on conditional mutual information and entropy, W Ya Di et al. proposed a new Max-Relevance and Min-Supervised Redundancy (MRMSR) criterion. It selects a specific set of features for each class tag, and proposes class tag specific mutual information. This method combines supervised similarity measurement with feature redundancy minimization evaluation. Then it is combined with feature correlation maximization evaluation. This solution lacks steps to eliminate redundant data [13].

Z Ping et al. first distinguished three types of label relationships, namely label independence, label redundancy, and label supplementation. After analyzing the changes and differences in label relationship based on different features, two new methods are proposed. One is Label Supplementation for Multi-Label FS (LSMFS). The other is Multi-label FS considering Maximum Label Supplementation (MLSMFS). This classification method cannot handle composite data well [14]. C Heng et al. proposed a simple and effective method called Unsupervised Feature Selection with Separability (UFS2). This method selects both feature and clustering data simultaneously. Binary vectors are seamlessly integrated into K-means to select an accurate feature for clustering. The parameter *k* (i.e. the number of selected features) is explicitly used. A customized binary vector term is designed to maximize the separation between the selected feature dimensions. It is not conducive to subsequent experimental follow-up [15].

S F Gharehchopogh et al. proposed an enhanced mutation operation algorithm based on binary multi-objective dynamic HHO and applied it to IoT botnet detection. The results show that the MODHHO algorithm has more advantages in cost than similar methods. Compared with other comparison algorithms, the MODHHO algorithm performs better on all five datasets. Compared with the proposed model machine learning methods on all five datasets, its error rate is lower according to the AUC, G-mean, and TPR standards [16]. S F Gharehchopogh et al. proposed an improved African vulture optimization algorithm for multi threshold image segmentation. Tests show that the algorithm achieves a good balance between exploration and utilization stages, avoiding getting stuck in local optima and providing high-quality segmentation solutions. Compared with other metaheuristic algorithms, this algorithm significantly improves its performance [17]. Özbay et al. proposed an improved ResNet50 convolutional neural network model for detecting acute lymphoblastic leukemia and its sub-types, which is hybridized through particle swarm optimization. Research shows that this method can help laboratory workers distinguish different types of acute lymphoblastic leukemia, and determine corresponding diagnostic and treatment plans [18]. S F Gharehchopogh et al. proposed an improved asymmetric self-organizing mapping asymmetric clustering method. The results show that the improved algorithm based on Chebyshev chaotic function is superior to other chaotic iteration and metaheuristic algorithms, which significantly improves the fitting and convergence speed [19]. S F Gharehchopogh proposed several improved Harris Eagle optimization algorithms for community detection in social networks. Research showed that this algorithm has good adaptability and effectiveness in identifying node structure positions and communities in social networks [20].

S F Gharehchopogh et al. proposed an emerging metaheuristic algorithm called "slime mold algorithm" (SMA) for research and analysis. According to the results statistics, by 2020, research based on SMA has been published in major scientific and technological databases, covering four fields: hybridization, development, deformation, and optimization of SMA algorithms, with the most widespread application in optimization problem solving [21]. M Ayar et al. proposed a novel feature selection method based on chaos partitioning for rapid automatic identification of arrhythmias. According to the results, on the arrhythmia dataset, the improved method removes redundant and noisy features while ensuring no information loss, significantly improving classification performance, achieving recognition accuracy of 88.21%. The diagnostic time is reduced by approximately 0.6s, which is a significant improvement compared with existing methods [22]. J Piri et al. proposed a novel feature selection method based on discrete artificial gorilla optimization for disease diagnosis in the field of healthcare. The results show that the mixed dual objective filter wrapper has the best performance. Compared with other filtering packaging methods, the initialization strategy of this method significantly improves population diversity and convergence speed. In multiple medical dataset comparison experiments, the classification performance of this method is significantly better than existing multi-objective feature selection algorithms. Finally, in the COVID-19 case experiment, this method successfully extracts relevant key diagnostic factors, demonstrating strong practical application value [23].

In conclusion, with the increase of data volume and complexity, it is more important to constantly optimize the FS algorithm. Many scholars have proposed a variety of methods, including weighted conditional mutual information FS method, uncertainty change rate FS method, multi label FS method, unsupervised FS method, etc. However, the effect is still difficult to achieve the desired state. Therefore, an optimized GA is proposed for high-dimensional data feature processing. The comparison of literature content is shown in Table 1.

**Table 1:** Comparison of literature content.

Method / Algorithm	Key Characteristics	Limitations	References
Weight Composition of Feature Relevance (WCFR)	• Based on weighted conditional information. • Uses SD to adjust importance between -Correlation and redundancy. • More effective without added time complexity.	May not fully address redundancy.	[9] H. Zhou, X. Wang, and Y. Zhang, "Feature selection based on weighted conditional mutual information," Appl. Comp. Inform., vol. 7, Jan. 2020, 10.1016/j.aci.2019.12.003.
FS algorithm of uncertainty change rate	• Considers uncertainty of class label reduction. • Accounts for increasing redundancy as more features are selected. • Complex calculation form.	Potential errors in measuring uncertainty.	[10] P. Zhang, and W. Gao, "Feature selection considering uncertainty change ratio of the class label," Appl. Soft Comput., vol. 95, no. 4, Oct. 2020, 10.1016/j.asoc.2020.106537.
Multi tag FS method for stream tagging	• Selects features based on Mutual information and discrimination index. • Produces final feature subset for each marker.	Subset may not label accurate information features.	[11] J. Liu, Y. Li, W. Weng, J. Zhang, B. Chen, and S. Wu, "Feature selection for multi-label learning with streaming label," Neurocomputing, vol. 387, pp. 268–278, Apr. 2020, 10.1016/j.neucom.2020.01.005.
Class-label specific mutual information (CSMI)	• Uses mutual information to correlate features and labels. • Aims to maximize shared information with the target class label.	Ignores information processing and targeted optimization of FS algorithm.	[12] D. K. Rakesh, and P. K. Jana, "A general framework for class label specific mutual information feature selection method," IEEE Trans. Inform. Theory, vol. 68, no. 12, pp. 7996–8014, Dec. 2022, 10.1109/TIT.2022.3188708.
Max-Relevance and Min-Supervised Redundancy (MRMSR)	• Based on conditional Mutual information and entropy. • Aims for supervised similarity measurement and feature redundancy minimization.	Lacks steps to eliminate redundant data.	[13] Y. Wang, X. Li, and R. Ruiz, "Feature selection with maximal relevance and minimal supervised redundancy," IEEE Trans. Cybern., vol. 53, no. 2, pp. 707–717, Feb. 2023, 10.1109/TCYB.2021.3139898.
LSMFS & MLSMFS	• Differentiates label relationships. • Proposes methods like LSMFS and MLSMFS for multi-label FS.	Struggles with composite data.	[14] P. Zhang, G. Liu, W. Gao, and J. Song, "Multi-label feature selection considering label supplementation," Patt. Recog., vol. 120, Dec. 2021, 10.1016/j.patcog.2021.108137.
Unsupervised FS with Separability (UFS2)	• Combines feature selection with clustering. • Integrates binary vectors into K-means. • Uses parameter k explicitly.	Not conducive to experimental follow-up and measurement optimization.	[15] H. Chang, J. Guo, and W. Zhu, "Rethinking embedded unsupervised feature selection: a simple joint approach," IEEE Trans. Big Data, vol. 9, no. 1, pp. 380–387, Feb. 2023, 10.1109/TBDATA.2022.3178715.
Various algorithms for optimization and detection	• Enhanced mutation in HHO for IoT botnet detection. • Improved African vulture optimization and other algorithms for varied applications.	-	[16] F. S. Gharehchopogh, B. Abdollahzadeh, S. Barshandeh, and B. Arasteh, "A multi-objective mutation-based dynamic Harris Hawks optimization for botnet detection in IoT," Internet of Things, vol. 24, pp. 100952–100962, 2023. https://doi.org/10.1016/j.iot.2023.100952.
FS method based on chaos partitioning	• Removes redundant and noisy features without information loss. • Improves classification performance and reduces diagnostic time.	-	[22] M. Ayar, A. Isazadeh, F. S. Gharehchopogh, and M. Seyedi, "Chaotic-based divide-and-conquer feature selection method and its application in cardiac arrhythmia classification," The Journal of Supercomputing, vol. 2022, pp. 1–27. https://doi.org/10.1007/s11227-021-04108-5.
Discrete artificial gorilla optimization	• Hybrid filter wrapper approach. • Improves population diversity and convergence speed. • Extracts key diagnostic factors for diseases like COVID-19.	-	[23] J. Piri, P. Mohapatra, B. Acharya, F. S. Gharehchopogh, V. C. Gerogiannis, A. Kanavos, and S. Manika, "Feature selection using artificial gorilla troop optimization for biomedical data: A case analysis with COVID-19 data," Mathematics, vol. 10, pp. 2742–2752, 2022. https://doi.org/10.3390/math10152742.

Table 1 presents a comparison of different researchers in the field of Feature Selection (FS). Researchers have adopted different methods or algorithms, highlighting their main characteristics and limitations. The WCFR method proposed by Z Hong Fang et al. emphasizes the importance of weights and information conditions, but may not fully address redundancy. Z Ping et al. focus on changes in label uncertainty, but the computational complexity is high. L Jing hua et al. and D K. Rakesh et al. focused on the mutual information between flow labels and class labels, but there are shortcomings in information capture and optimization. W Ya Di et al. and C Heng et al. proposed methods from the perspectives of supervised and unsupervised learning, respectively. The methods of Gharehchopogh F S, Ayar M et al., and Piri J et al. focus on optimizing the efficiency and accuracy of algorithms and disease diagnosis, but the limitations of the latter two are not mentioned in the table.

## 3. FS algorithm based on optimized genetic algorithm and the modeling in high-dimensional data processing

The method of constructing and processing feature subsets is used to mine the optimal population. The improved GA-KNN is adopted to further improve the accuracy of the FS model. Meanwhile, various aspects of GA have been improved to find the optimal solution in complex high-dimensional data. The FS model of high-dimensional data processing is used to find the optimal solution among many features to achieve more accurate modeling and analysis.

### 3.1. Feature selection based on matrix structure genetic algorithm

By constructing a matrix structure, the FS problem is transformed into finding the optimal feature subset in the search space. The global search ability and fitness function guided optimization process of GA can identify the most representative and explanatory feature combinations from a wide range of possible solutions. Thus, more accurate predictions and understanding can be obtained when dealing with complex high-dimensional data models. The optimization function is used as the testing objective to obtain the effectiveness of the testing algorithm [24, 25]. The function is used to solve the optimal solution without distortion, as shown in Eq (1).


$$\left\{ \begin{array}{l} \min f(x)\\ subjecttox\in \Omega \end{array}\right.$$ (1)

**Fig 1:**
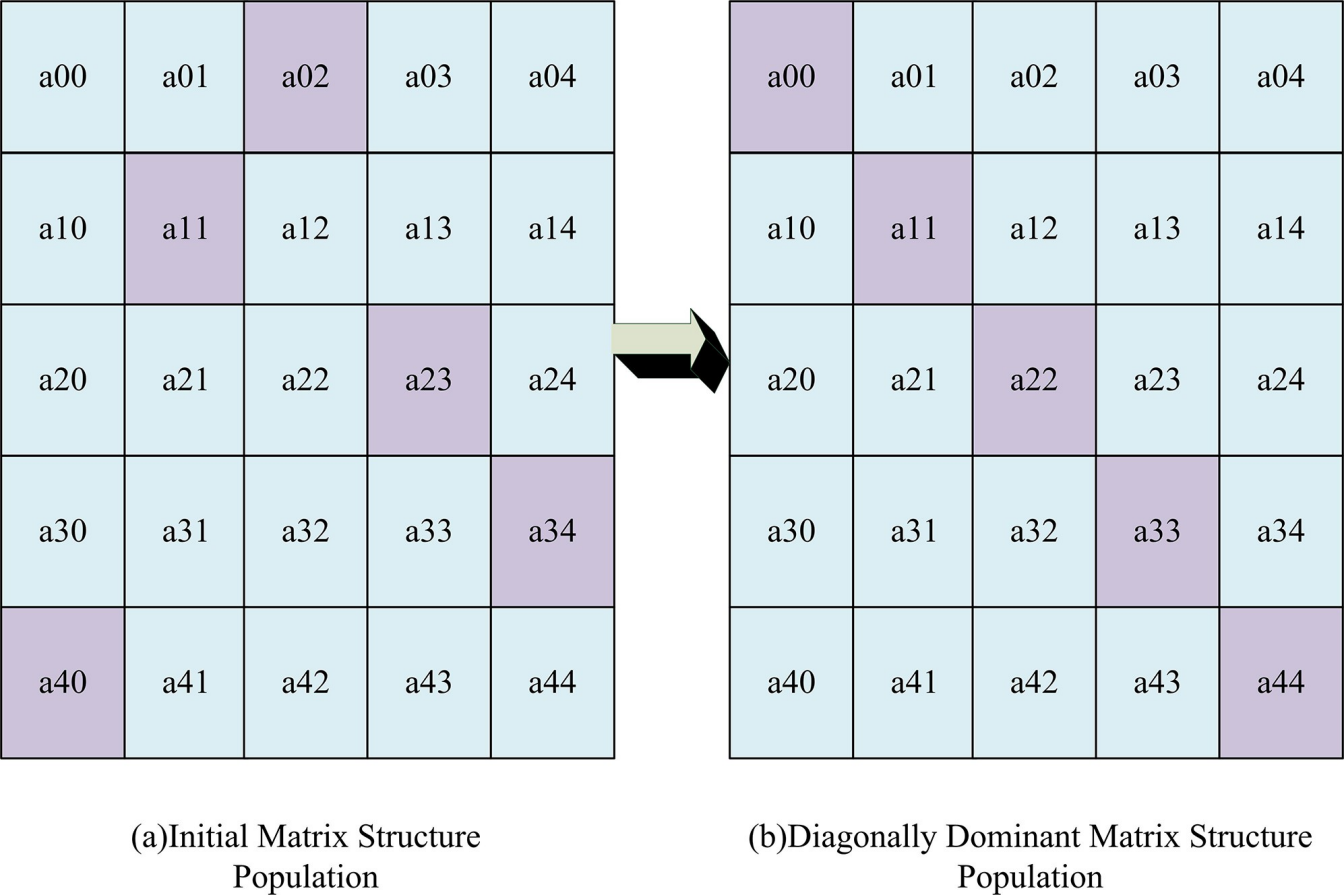
Diagonally dominant MGA and initial MGA comparison chart.

In Eq (1), *x* is the decision vector. *f*(*x*) is the objective function. Ω is the decision space. In standard GA, binary encoding is not ideal for function optimization. Therefore, the research direction gradually turns to Particle swarm optimization algorithm and differential optimization algorithm. Multiple iterations make it difficult to improve performance. The mutation factors that undergo multiple iterations also leads to gene degradation. Due to various reasons, GA has low computational efficiency and poor global convergence performance. Therefore, it is quite necessary to improve the GA. MGA is a population evolution algorithm organized by two-dimensional relationships. For population grouping, multiple one-dimensional populations form a two-dimensional population. The concept of main diagonal position is suitable for simple and convenient operator design. It is valuable for engineering implementation of genetic system [26]. The comparison diagram between diagonally dominant MGA and initial MGA is shown in Fig 1.

**Fig 2:**
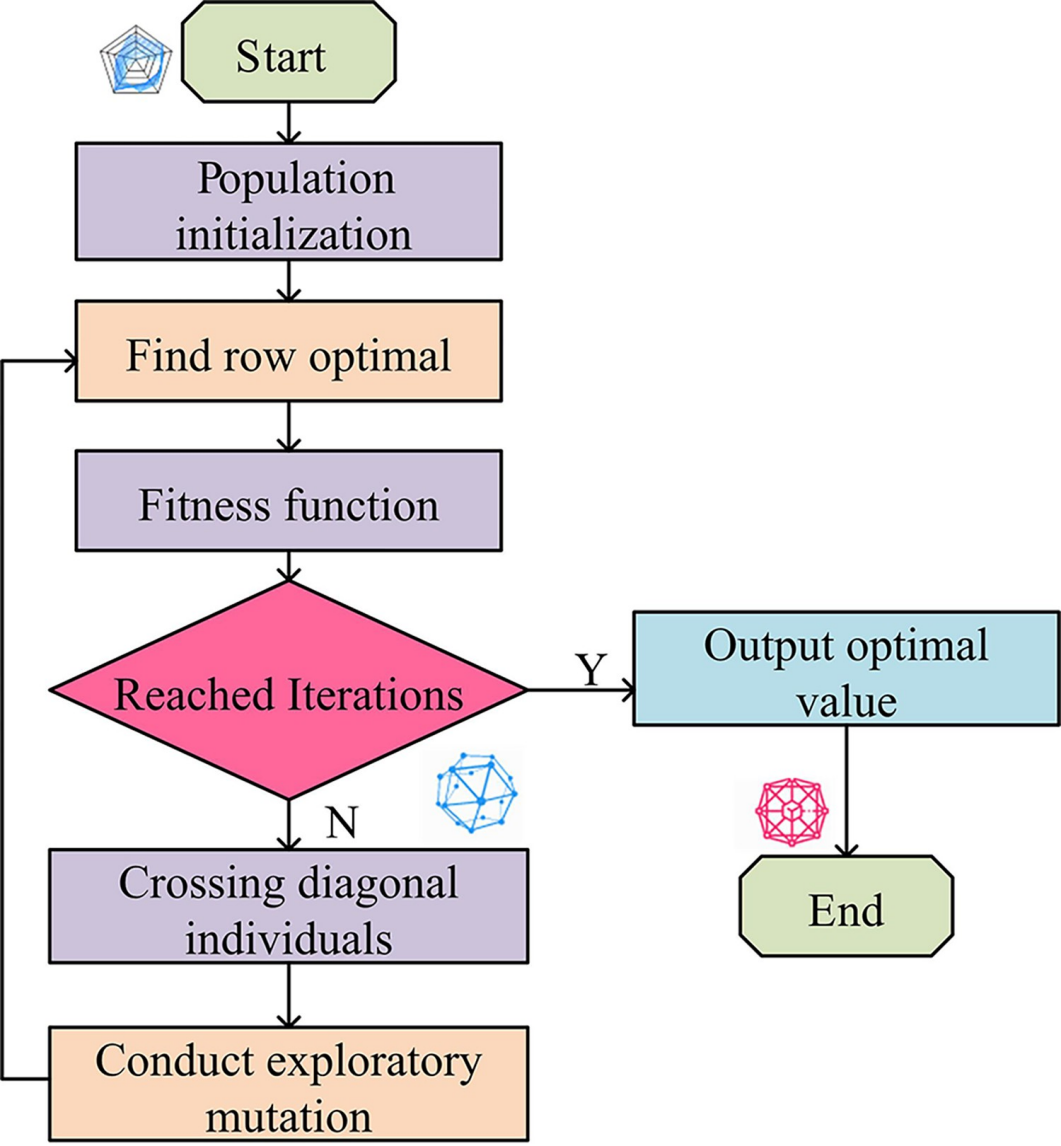
MGA algorithm flowchart.

In Fig 1(A), in a population structure of 25, the storage structure uses a two-dimensional array. Each element of an array is an individual object, representing a possible solution to the problem. In each row, the position where the optimal fitness value first appears is marked in gray. In Fig 1(B), the diagonal population with rows equal to columns is displayed. After the population structure is clarified, operators with different meanings can be defined accordingly. After completing this exploratory mutation process, a new generation population is formed and ready to enter the next round of evolution. These new individuals once again undergo a selection, crossover, and mutation cycle. This iterative process is always fitness oriented, ensuring that the excellent genes of outstanding individuals can be inherited and spread. Once the established termination criteria, such as the iterations or fitness values, are met, the iteration will stop. The final result is the optimal and highly adaptable individuals that have been optimized through multiple generations. This individual feature provides the best FS scheme to achieve more accurate modeling results in complex high-dimensional data structures. One or more genetic points will be selected and exchanged from individual parents to produce new offspring with better traits. The MGA flowchart is shown in Fig 2.

In Fig 2, the structural population is initialized. The size of each sub-population is determined. Then, the optimal elements are searched line by line. Then the population is empowered. In the main diagonal, the optimal individuals are searched and judged. The conditions are determined. Furthermore, the cross judgment is applied to different conditional elements and new meanings are given to newly generated individuals. In response to the new significance, the fitness values of temporary individuals are mutated and empowered. After constructing the initial population and individual definitions, a series of operations are carried out to optimize the population. By searching the optimal elements of each sub-population, the optimal individuals on the main diagonal are determined. The corresponding conditions are determined. Then, the elements that meet the conditions are subjected to cross determination. The newly generated individuals are assigned new attributes and meanings. To further improve population fitness, objective mutation operations are implemented. This process is repeated, forming an iterative cycle until the termination conditions are met, such as reaching a predetermined iterations or the expected value. An optimized and completely new population is generated, with a significantly higher fitness value than the initial population. In this way, the optimized population can be used to solve problems such as high-dimensional data FS, which can greatly improve the accuracy and efficiency of the model.

### 3.2. Feature selection based on improved GA-KNN algorithm

When solving the FS for high-dimensional data, the accuracy and efficiency of the algorithm are crucial. The improved GA-KNN feature selection method adopts an optimized GA. In the refining iteration process, the FS in the K-Nearest Neighbor (KNN) classifier is gradually optimized. Combining the global search characteristics of GA with the simplicity and intuitiveness of KNN, the goal is to find the most representative feature combination in the feature space, thereby optimizing the overall model performance. The training dataset of the K-nearest algorithm is shown in Eq (2).


$$T=\{\left(x_{1},y_{1}),(x_{2},y_{2}),\cdots ,(x_{N},y_{N}\right)\}$$ (2)

**Fig 3:**
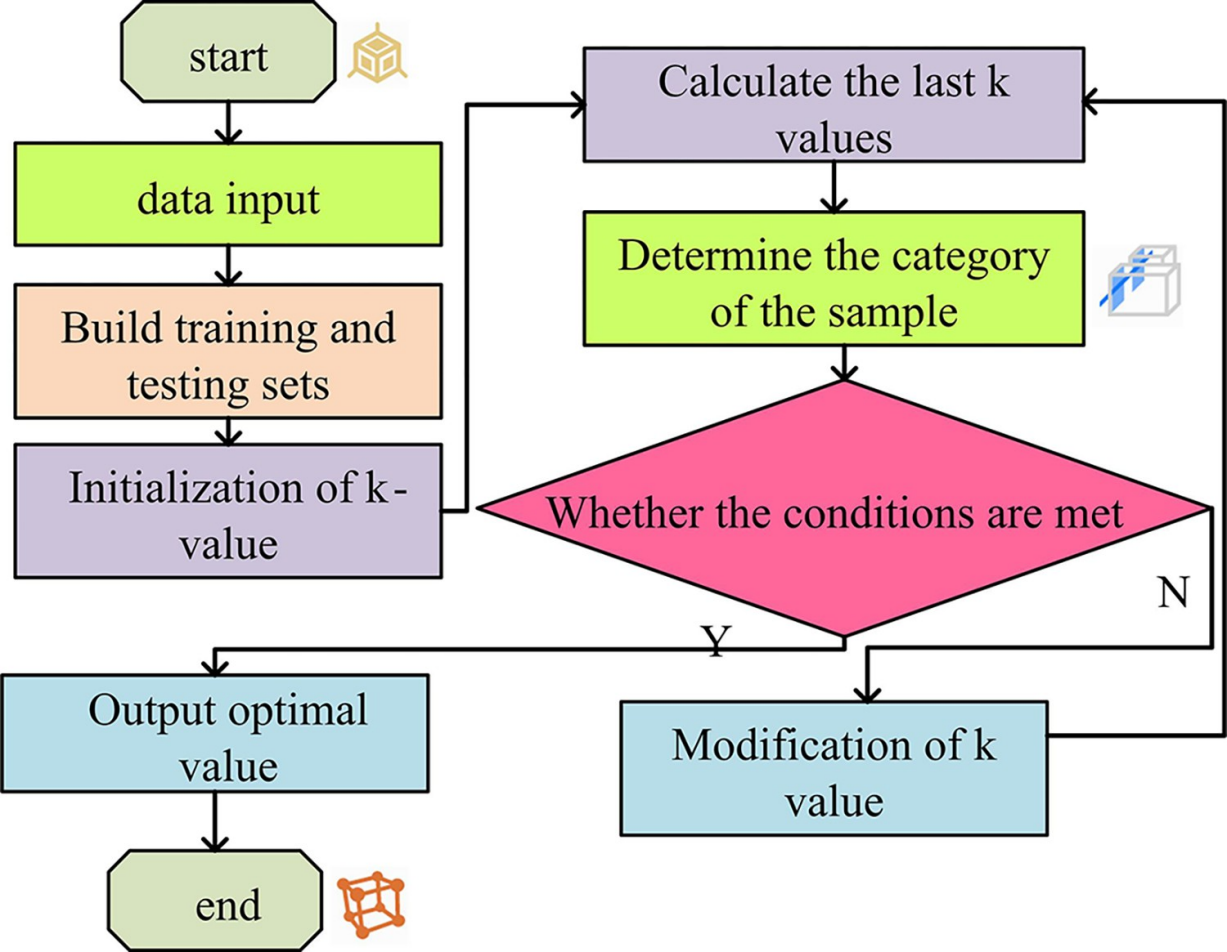
KNN algorithm flowchart.

In Eq (2), *x*_*i*_∈*X* is the feature vector of the instance. *y*_*i*_∈*Y* is the category of the sample, and *i* = 1,2,…,*N*. *x* is the feature vector of the sample. Fig 3 is the KNN flowchart.

In Fig 3, the collected data is selected for FS. The data source can be various databases. At the same time, the dataset consists of training and testing sets. The training set accounts for the majority. The data sample is constructed. A small amount of redundant data is used as test samples. Then the K value is set. The odd number is chosen for the K value. Furthermore, based on the distance equation, k points are found in the test sample and training set, forming a domain, denoted as *N*_*k*_(*t*). The classification decision planning in this field is shown in Eq (3).


$$y=\arg\;\underset{c_{j}}{\max}\sum_{x_{i}\in N_{k}(x)}I(y_{i}=c_{j}),i=1,2,\ldots ,N;j=1,2,\ldots ,K$$ (3)

In Eq (3), *I* is the indicator function. When the iterations or accuracy reach the optimal value, the loop stops. The optimal result of classification is output. There is a real number vector in the feature space. The Chebyshev distance is used to solve the problem, as illustrated in Eq (4).


$$L_{\infty }(x_{i},y_{j})=\underset{l}{\max}|x_{i}^{\left(l\right)}-y_{j}^{\left(l\right)}|$$ (4)

**Fig 4:**
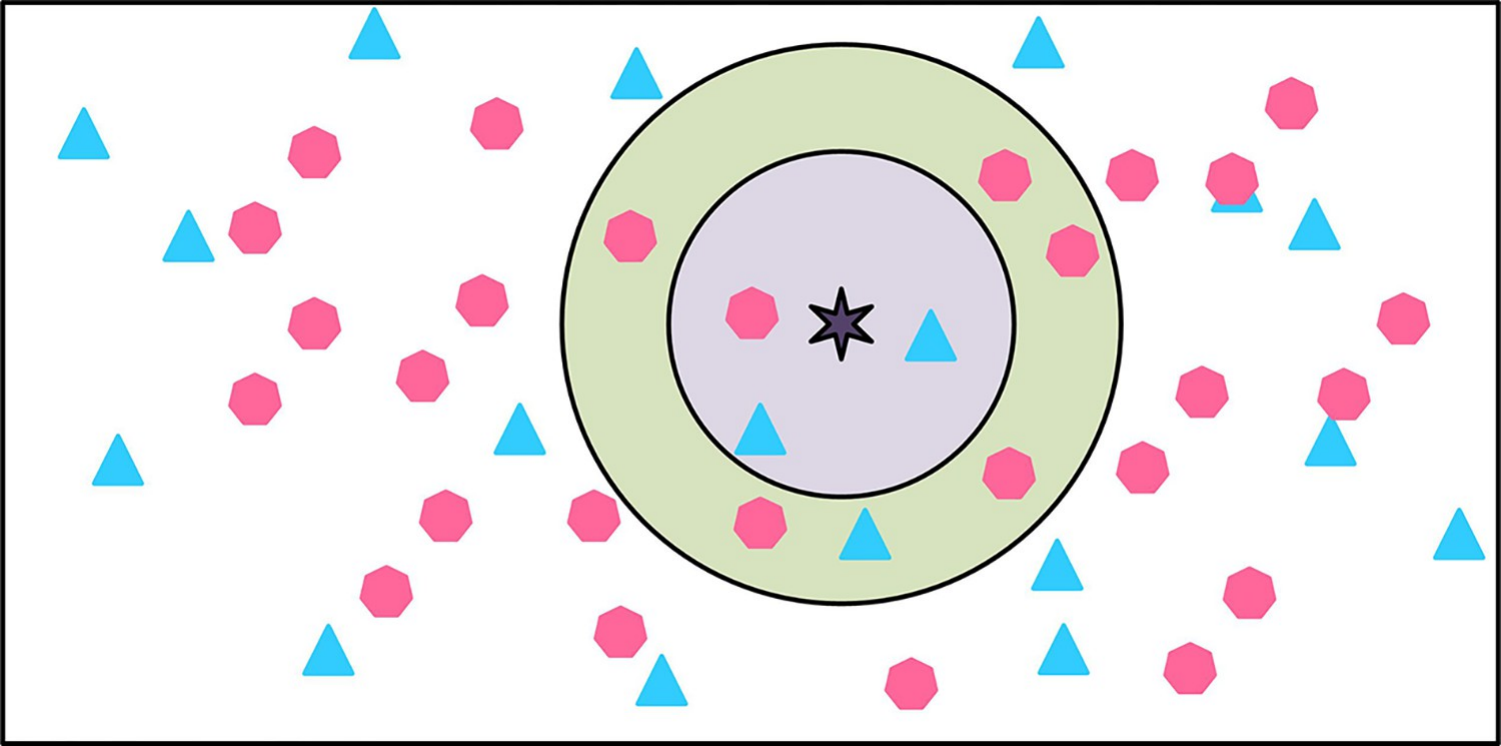
The impact of different k-value classifications.

In Eq (4), the k value is a crucial issue in the KNN algorithm, which greatly affects the final selection result. Therefore, when the k-value is chosen very small, a small number of training samples are used to predict the test samples. This results in a decrease in the approximation error of the training model. Only test samples that are closer to the input samples have impacts on the predicted results. The classification prediction diagram for different k values is shown in Fig 4.

In Fig 4, in the KNN algorithm, the k value has a significant impact on model performance. When the k is small, the model complexity is high. The training error is small, but it is easy to over fit. When the k-value is large, the model complexity decreases. The training error increases, but the generalization ability may be enhanced. The classification decision in the KNN nearest neighbor algorithm is obtained based on the rule that the minority follows the majority. The category of input sample individuals is determined by multiple classes from the nearest k training instances among the input sample individuals. According to the discrimination principle, the classification function is shown in Eq (5).


$$f:R^{n}\to \{c_{1},c_{2},\cdots ,c_{k}\}$$ (5)

In Eq (5), the Loss function is 0 to 1. Furthermore, the mis-classification probability is shown in Eq (6).


P(Y≠f(X))=1−P(Y=f(X)) (6)

In Eq (6), for a given training sample, individuals are combined into a set. If the covered area category is *c*_*j*_, the mis-classification probability is shown in Eq (7).


$$\frac{1}{k}\sum_{x\in N_{k}(x)}I(y_{i}\neq c_{i})=1-\frac{1}{k}\sum_{x\in N_{k}(x)}I(y_{i}=c_{i})$$ (7)

In Eq (7), the performance of a classification model can be measured by the mis-classification probability. For binary classification problems, the mis-classification probability can be defined as the probability that the model misjudges positive cases as negative cases, or misjudges negative cases as positive cases. The distance between two individuals is shown in Eq (8).


$$L_{p}\left(x_{i},x_{j}\right)={\left(\sum_{l=1}^{n}\left|w_{i}x_{i}^{\left(l\right)}-w_{i}x_{i}^{\left(l\right)}\right|\right)}^{\frac{1}{p}}$$ (8)

In Eq (8), *L*_*p*_ is the distance between two individuals. "Taxicab geometry" is used for calculation, as shown in Eq (9).


$$d\left(x_{i}^{*},x_{j}^{*}\right)=\frac{\sum_{n=1}^{N}Z_{in}Z_{jn}|x_{in}^{*}-x_{jn}^{*}|}{\sum_{n=1}^{N}Z_{in}Z_{jn}}$$ (9)

In Eq (9), $x_{i}^{*}$ is the sample to be valued. When *x*_*in*_ and *x*_*jn*_ have no outlier, $x_{in}^{*}$ and $x_{jn}^{*}$ are calculated into $d(x_{i}^{*},x_{j}^{*})$. The missing values for data standardization are estimated, as shown in Eq (10).


$${\hat{x}}_{in}^{*}=\sum_{{\hat{x}}_{j}^{*}\in \theta_{i},Z_{jn}=1}\beta_{ij}x_{jn}^{*}$$ (10)

In Eq (10), the *K* adjacent set *θ*_*i*_ of $x_{i}^{*}$ is determined by the Taxicab geometry. As a result, the distance related weights are calculated. Finally, further calculations are made, as shown in Eq (11).


$${\hat{x}}_{in}={\hat{x}}_{in}^{*}\cdot \sigma_{n}+\mu_{n}$$ (11)

**Fig 5:**
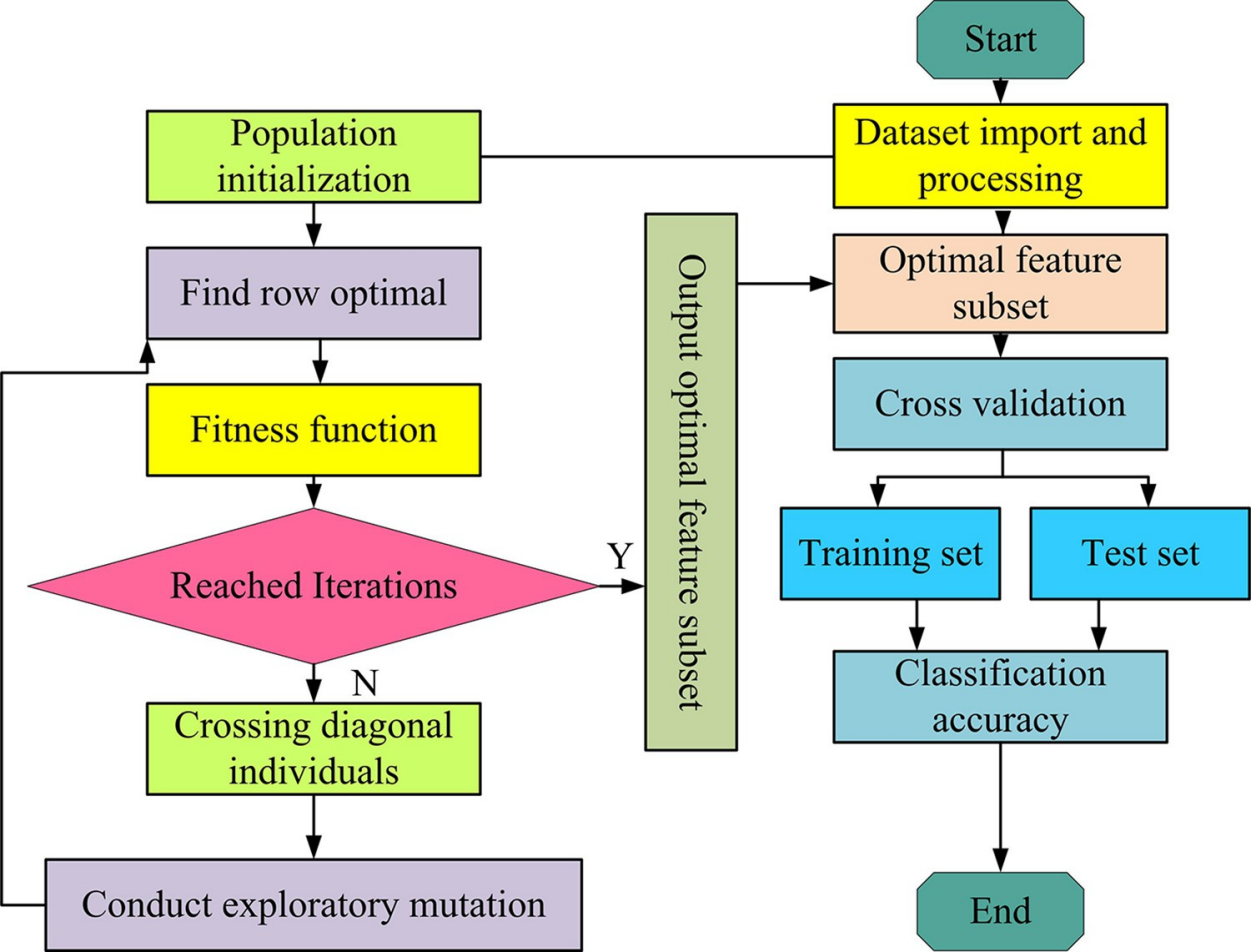
GA-KNN algorithm flowchart.

In Eq (11), $x_{in}^{*}$ is the standardized value. ${\hat{x}}_{in}$ is the missing value after affine transformation and further estimation. The availability optimization method based on KNN is simple and effective. However, the valuation accuracy is not high. Therefore, iterative data based on orthogonal Matching pursuit is required. Furthermore, the GA is introduced to construct the GA-KNN algorithm. The process is shown in Fig 5.

In Fig 5, firstly, a dataset is imported. The empowerment method is used for weight assignment. Then the dataset is encoded to initialize the population. Next, GA is used to carefully screen, cross pair, and mutate each individual in the population to optimize these individuals in each one. In addition, the classification accuracy of each individual is calculated as the fitness function value. As long as the preset maximum number of iterations is reached, the individual with the highest fitness value is selected. It is the optimal feature subset. Finally, to further verify the effectiveness of the selected feature subsets, these feature subsets are applied to the dataset for testing and verification. By calculating fitness function and ranking them according to their fitness size, they have the optimal priority. Therefore, in GA, the fitness selection directly affects the optimization speed. To gain a more objective understanding for the effectiveness of the used method, accuracy is used as a criterion for classification evaluation. The indicator is shown in Eq (12).


$$Accuracy=\frac{TP+TN}{TP+TN+FP+FN}$$ (12)

In Eq (12), *TP* is the number of positive classes for positive class prediction. *TN* is the number of negative classes for negative class prediction. *FN* is the negative class number for positive class prediction. *FP* is the positive number for negative class prediction.

### 3.3. Construction of FS algorithm model integrating high-dimensional data processing with optimized genetic algorithm

Chi square test is a statistical method used to evaluate the correlation between features and target variables. It works by comparing the difference between the actual observed frequency and the expected frequency. For each feature in the dataset, this method calculates a chi square statistic to evaluate its strength of association with the target variable. The larger the statistic, the stronger the correlation between the feature and the target variable.

**Fig 6:**
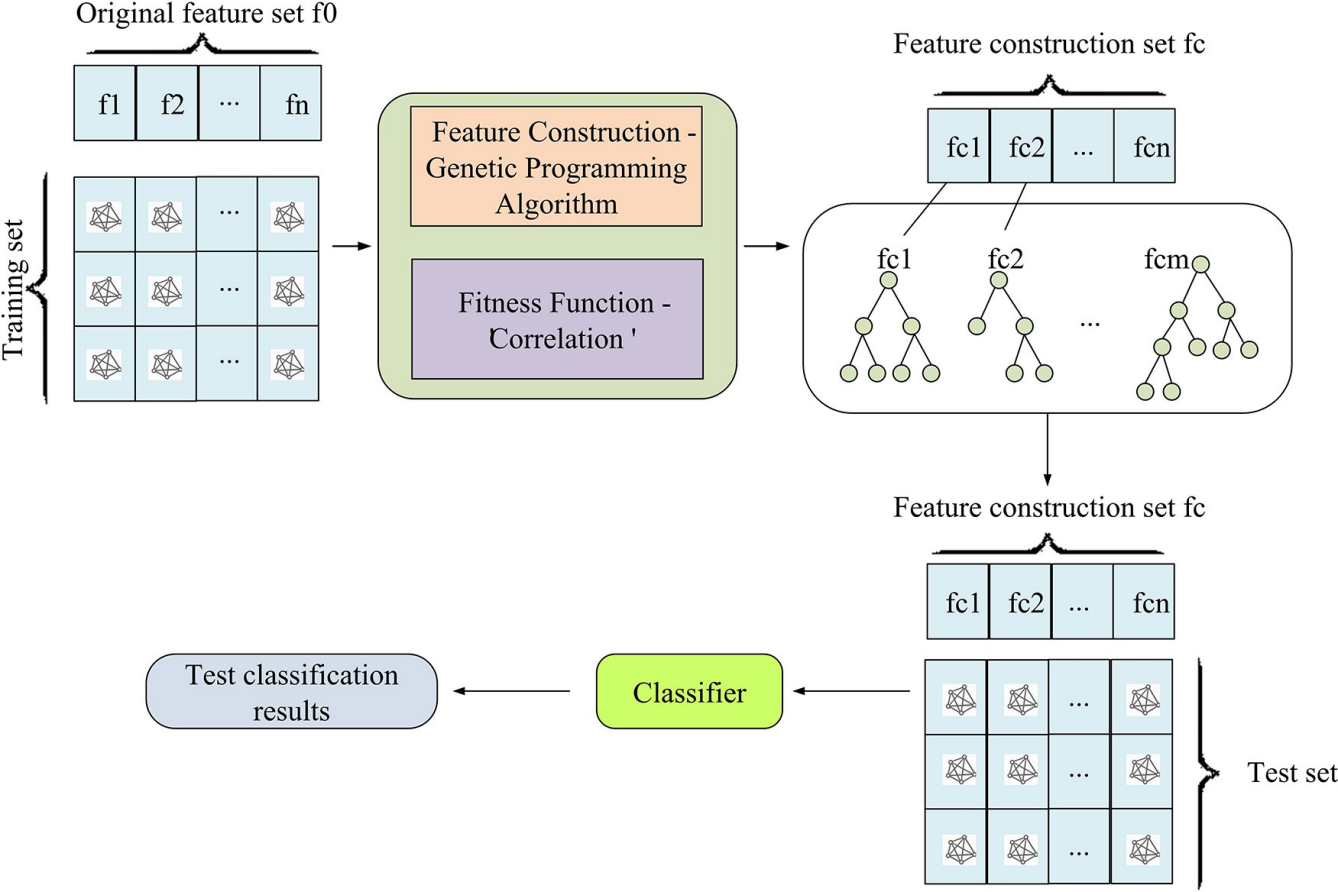
Run chart of multi feature construction method.

The optimized GA is integrated into the processing model of high-dimensional data. The global search ability and self-adaptability of the GA provide effective means for high-dimensional data processing. The first task is to understand this optimized GA in detail, and then explain the application in feature selection. Subsequently, a complete model with the ability to process high-dimensional data is constructed. The general feature selection process can only screen out relatively valuable features from the original features. But it is unable to deeply explore the interactions between features. Therefore, some predictive models may not exhibit excellent predictive performance in certain scenarios. Feature combination can combine multiple related features together to generate a new feature, which helps to reveal the possible joint or interactive relationships between features. Feature construction is to create new features based on existing features through some calculation methods, which can mine more information hidden in the original data. For example, feature construction can be carried out through statistical analysis, complex models such as deep learning, and other means. For this problem, the feature is constructed based on the completed FS, creating a new feature. The constructed multi feature method is shown in Fig 6.

In Fig 6, after running a single feature construction multiple times, the optimal construction individual obtained each time is returned to establish a feature set containing the optimal construction individual. The hybrid method of feature selection and feature construction is applied to perform FS on the original dataset to obtain the optimal feature subset. During the feature construction process, multiple features are constructed based on the selected feature subset. Then, through classifier classification testing, it is verified whether the constructed features can improve classification accuracy. The feature construction and selection of the GA are similar. The difference lies in the subset of features output. The output of feature selection is the terminal node of the individual tree. The output of feature construction is the entire tree operation result or expression. The Chi-square validation method is to sort by calculating the Chi-square values of each feature. The Chi-square value is directly proportional to the correlation. A small Chi-square value indicates a low correlation. On the contrary, if the Chi-square value is relatively large, then the correlation is also relatively large, as shown in Eq (13).


$$x^{2}=\sum_{i=1}^{k}\frac{{\left(f_{i}-np_{i}\right)}^{2}}{np_{i}}$$ (13)

**Fig 7:**
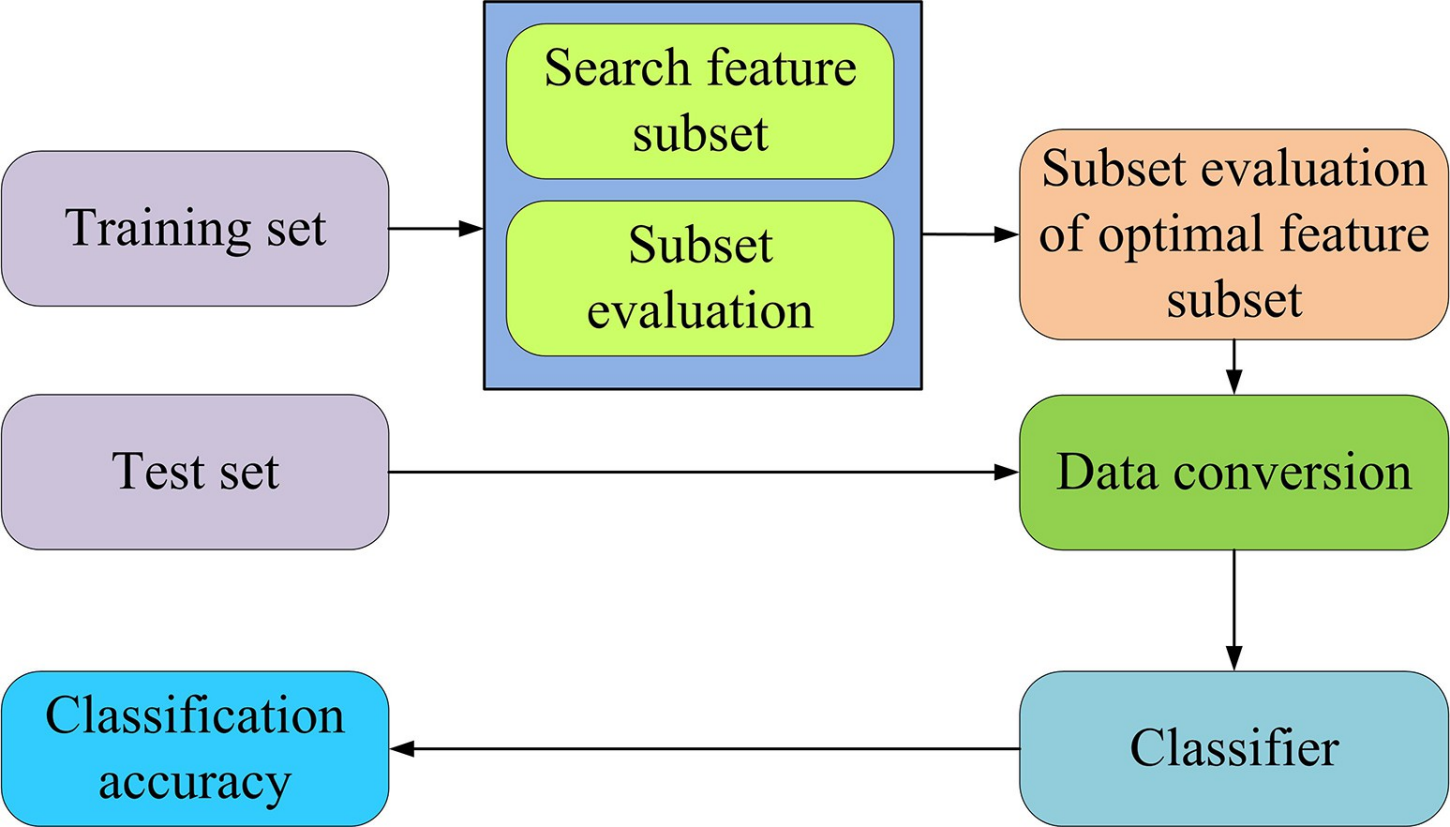
Run chart of multi feature construction method.

In Eq (13), *f*_*i*_ is the horizontal observation frequency. *n* is the total frequency. *p*_*i*_ is the expected frequency. In the classification, the training set is used to select the optimal subset of features. After feature transformation, the newly generated test set is classified on the classifier for effectiveness. Fig 7 illustrates the classification flowchart.

In Fig 7, firstly, the training set is applied to select the optimal feature subset and perform feature conversion. This step aims to capture and extract important information in the original data. After this processing step, a new test set is generated. Next, the new test set is placed into the pre-trained classifier for classification tasks. This classification process can be clearly presented using a flowchart. The left part of the flowchart represents the training set preparation and the feature subset selection, followed by the feature transformation steps. After the conversion is completed, a new test set is generated and entered into the classifier for classification. On the right side of the flow chart, the final classification results can be obtained, from optimizing feature selection to feature conversion, and then to the final classification stage. Each step determines the quality of the final classification result, which plays a crucial function in the machine learning process.

The FS algorithm combines the optimization genetic algorithm and high-dimensional data processing, which has low computational complexity when dealing with a large number of features, and avoids the premature convergence and scalability problems of traditional genetic algorithm in high-dimensional space. The optimization algorithm uses matrix structure and diagonal dominant algorithm to reduce the search space, accelerate convergence, and achieve polynomial time complexity. Compared with exhaustive search and filter methods, it is more efficient on large data sets. Combined with k-nearest neighbor classifier, it refines feature selection through iterative search, maintains a balance between exploration and utilization, and the combination of chi-square verification does not increase the computational burden. This improves model parsimony and computational efficiency.

## 4. FS algorithm based on optimized GA and the application in high-dimensional data processing

The FS algorithm based on optimized GA has important application value in high-dimensional data processing. Based on the GA optimization mechanism, the algorithm can select the most relevant and representative feature subset from numerous features, thereby reducing dimension and enhancing the accuracy.

### 4.1. Feature selection test results based on the matrix structure and improved GA-KNN algorithm

The study is evaluated using 80000 training sets and 15000 test sets from the Fashion-MNIST database. After the weight distribution of coding features, the improved GA is applied to find the optimal feature subset. In this process, the GA evaluates each candidate solution, which is a subset of features. New candidate solutions are generated through selection, crossover, and mutation operations. After finding the optimal feature subset, the KNN algorithm is used to classify them. In the KNN algorithm, an unlabeled sample is assigned to the class in which its K nearest neighbors are most common. The classification performance of KNN algorithm is compared when selecting all features and only the optimal subset of features to evaluate the effectiveness of the feature selection method. The parameterized environment is shown in Table 2.

**Table 2:** Parametric environment.

Hardware/Software	Specification	Remarks
CPU	Intel® Core™ i7-8700K CPU @ 3.70GHz	Provides excellent computational ability, ensuring efficient data processing and model training
RAM	64GB DDR4	Large memory capacity ensures that it can withstand a large amount of computation and parallel processing tasks when processing high-dimensional data
GPU	Nvidia GeForce GTX 1080 Ti	Equipped with dedicated deep learning computing cores, helpful in accelerating the calculation process of genetic algorithms and KNN algorithms
Hard Disk	2TB SSD	Provides ample storage space to store the large-scale Fashion-MNIST dataset and intermediate results
Operating System	Ubuntu 18.04 LTS	A stable and reliable operating system, widely used in the field of scientific computing
Programming Language	Python 3.8	Rich scientific computing and machine learning libraries, widely used in data science and machine learning tasks
Data Processing and Computing Libraries	Numpy 1.20.1 and Pandas 1.2.4	Provide powerful data processing and computing capabilities
Machine Learning Library	Scikit-learn 0.24.1	Used to implement KNN algorithm and evaluate the model
Graphics Visualization Library	Matplotlib 3.3.4	Used for creating the convergence result visualization in Fig 8

Table 2 describes a parameterized environment, including hardware and software specifications and their notes. In terms of hardware, it is equipped with Intel ® Core ™ A high-performance computing platform with i7-8700K CPU and 64GB DDR4 memory, as well as Nvidia GeForce GTX 1080 Ti GPU and 2TB SSD hard drive, ensures data processing capability and storage space. In terms of software, the Ubuntu 18.04 LTS operating system and Python 3.8 programming language are used, supplemented by Numpy, Pandas, Scikit learn, and Matplotlib libraries to process data, implement machine learning algorithms, and visualize graphics.

**Fig 8:**
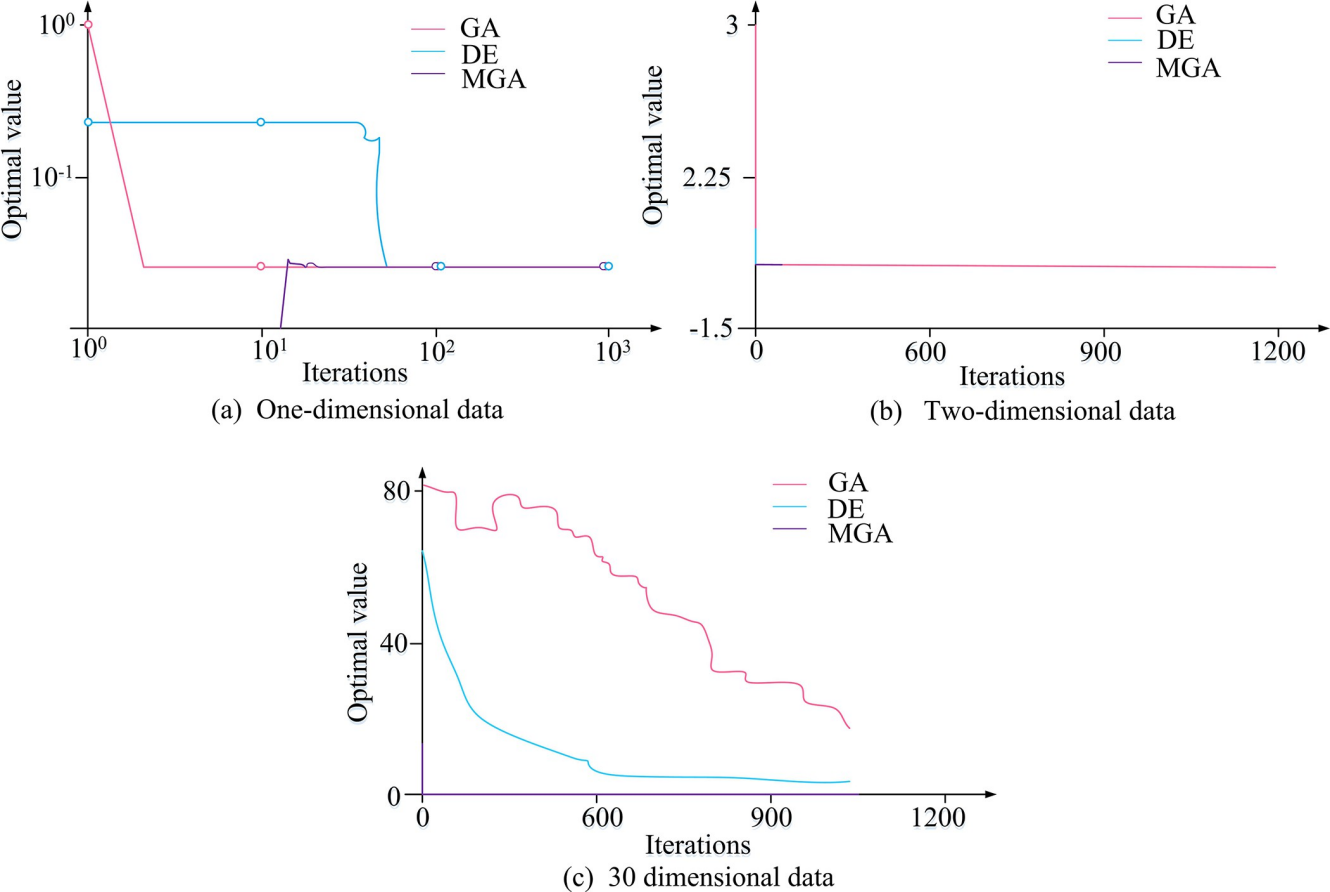
Convergence results of different dimensional functions under different algorithms.

For functions with different dimensions, there are 1, 2, 3,…, 30 dimensional function. Some dimensions are intercepted for analysis, as shown in Fig 8.

**Fig 9:**
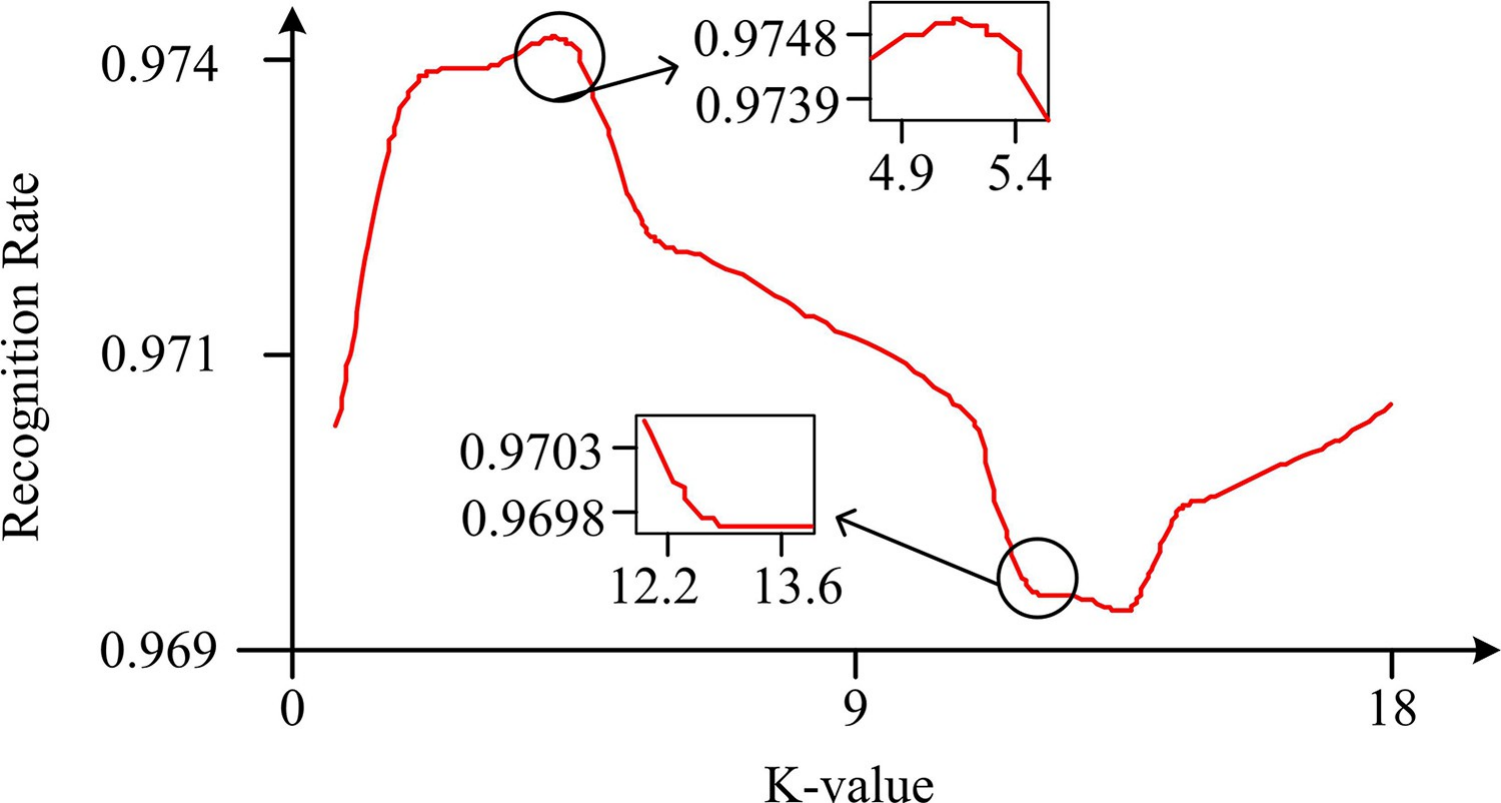
Change curve of classifier recognition rate and k-value.

In Fig 8, in the one-dimensional convergence data, the optimal value of GA decreases from 101.5 to 100.5 when the iteration reaches 101.6. The optimal value of Differential Evolution (DE) has a fault between 100.2 and 101.1 iterations, and then stabilizes at 100.5. The Multi-Objective Genetic Algorithm (MGA) has the least number of iterations and wastes the least time. In the two-dimensional convergence data, GA has 0 iterations at an optimal value of -0.5, while MGA has the 0 iteration at an optimal value of 3. DE has the optimal value of 1 in 0 iterations. However, after iteration, the optimal values of these three algorithms stabilize at -1. In the 30-dimensional convergence data, MGA has the best iteration efficiency. Meanwhile, a high dimension indicates poor convergence performance of DE and GA. 80000 training sets and 20000 test sets from the Fashion-MNIST database are used for evaluation. After assigning weights to encoding features, an improved GA is applied to find the optimal feature subset. Then the KNN classification algorithm is adopted for testing. The relationship between k-value and accuracy can be used to obtain the change curve of classifier recognition rate and k-value, as shown in Fig 9.

In Fig 9, when the K value is 7, the classification effect is optimal. The recognition level corresponding to different K values is used to determine the size of K. When K is less than 4 or greater than 8, the corresponding recognition rate is highly low. When K is between 4 and 8, the corresponding recognition difference is small. The consistency and stability of this choice are confirmed through extensive cross validation testing on different datasets. Statistical analysis is conducted to ensure that the improvement in recognition rate at K = 7 is not a random coincidence, but a significant upward trend in classification performance. In addition, the data set used covers different fields and sizes, indicating that K = 7 is a robust selection unaffected by data diversity. According to the sensitivity analysis, at K = 7, the system achieves a balance between under-fitting and over-fitting, indicating that the model complexity is very suitable for underlying patterns in data. Therefore, when K is 7, the best classification performance can be achieved. Table 3 displays the corresponding GA-KNN feature selection experiment results.

**Table 3:** The number of features and classifier recognition rate in different weights.

Encoding method	Before feature selection	Correct recognition rate	After feature selection	Correct recognition rate
Binary encoding	785	0.9694	410	0.9688
Decimal code	728	0.9596	295	0.9814
Real code	700	0.9725	356	0.9681
Gaussian code	714	0.9625	406	0.9754
Adaptive encoding	724	0.9352	372	0.9815

In Table 3, when different encoding methods assign weights to features, the features are greatly reduced. The feature recognition rate has been improved, indicating that assigning weights to the sample data is effective. The correct recognition rate of binary encoding has a deviation of 0.0006 before and after. The decimal coding accuracy is 0.0218. The adaptive bias is filtered from 724 to 372. The accuracy has increased from 0.9352 to 0.9815. After screening from 714, only 406 Gaussian codes are retained. The accuracy increases from 0.9625 to 0.9754.

### 4.2. Feature selection analysis of high-dimensional data processing integrated with optimization algorithm

Eight high-dimensional data sets are used for experiments. These eight datasets have different feature numbers, sample sizes, and categories. The feature dimension ranges from several thousand to ten thousand. However, these data sets have a common characteristic, which has very high feature dimensions and few individuals. Compared with thousands of feature dimensions, most data sets have fewer than 100 individuals. In addition, these data sets are used to research feature selection and feature construction, which is representative. This type of data generally has high latitude, high redundancy, few samples, and imbalanced samples. When the GA runs, the population initialization process is random. To make the experimental results more accurate, each data set is randomly divided into different training and testing sets, with 70% being used as the training set and 30% as the testing set. During the experiment, each method is run 50 times on both the training and testing sets. Multiple experiments are conducted to make the experimental results more convincing and reduce result bias caused by data set segmentation or genetic programming randomness. Table 4 illustrates the parameter settings used in the experiment.

**Table 4:** Data set and experimental parameters.

Data set	Characteristic number	Number of samples	Number of classes	Parameter	Parameter value
Colon	1800	70	3	Function set	+、−、×、÷
SRBCT	2200	80	5	Terminal set	Characteristic
Lymphoma	7100	60	4	Maximum tree depth	19
Leukemia	7100	70	3	Generation	55
Leukemia_3c	7100	70	3	Population size	600
CNS	7100	50	3	Crossover rate	1.0
MLL	12500	70	2	Selection method	Championship selection method
Ovarian	15100	260	4	Rproduction rate	0.2
Lung	8000	100	3	Mutation Rate	0.2
Kidney	9000	120	3	Function Set	+, -, *, /
Liver	10000	140	4	Terminal Settings	Feature
Stomach	11000	160	3	Maximum Tree Depth	21
Skeleton	13000	180	3	Generation	60
Red Blood Cells	7500	200	5	Population Size	700

In Table 4, Colon, Leukemia, CNS, Ovarian represent the binary dataset, while SRBCT, Lymphoma, Leukemia 3c, Ovarian represent the multi-class dataset. Colon, CNS, Ovarian include two categories, namely the diseased category and the normal category. Leukemia includes acute lymphocytic leukemia and acute myeloid leukemia. The SRBCT dataset contains four cancer categories, namely Ewing sarcoma, non Hodgkin lymphoma, *Neuroblastoma* and *Rhabdomyosarcoma*. The MLL dataset contains three categories, namely acute lymphoblastic leukemia, acute myeloid leukemia, and mixed lineage leukemia. The Leukemia 3c dataset contains three categories, namely B-ce11, T-ce "and AML. The three disease categories in the Lymphoma dataset are diffuse large B-cell lymphoma, Chronic lymphocytic leukemia and non Hodgkin lymphoma. For classifiers, a week package is used for adjustment and evaluation, with each classifier running 60 times. The average accuracy rate, maximum accuracy rate, and average feature count of these 60 attempts are counted. Finally, the average accuracy is used as an indicator. The maximum tree depth is 19. The sensitivity analysis is shown in Table 5.

**Table 5:** Sensitivity analysis of the proposed algorithm.

Parameter	Initial Value	Modified Value	Accuracy Change	Feature Count Change	Remarks
Function Set	Default	Extended	-3%	+15	Extension led to slight overfitting
Terminal Set	Features	Extended Features	+5%	-10	Enhanced feature representation improved accuracy
Maximum Tree Depth	19	10	-2%	-5	Shallower trees reduced complexity but also accuracy
Generations	55	100	+4%	+8	Increased generations enhanced model stability
Population Size	600	300	-1%	+20	Reduced population sped up computation with a slight accuracy drop
Crossover Rate	1.0	0.8	+2%	-7	Lower crossover rate increased diversity
Selection Method	Tournament	Roulette Wheel	-2%	+5	Roulette Wheel reduced selection precision
Reproduction Rate	0.2	0.4	+1%	-3	Higher reproduction rate improved diversity of solutions
Mutation Rate	0.2	0.1	-1%	+12	Lower mutation rate reduced excessive random search

In Table 5, by adjusting the algorithm parameters, the trends of different indicators can be observed [27]. Function set expansion may lead to over-fitting, resulting in a decrease in accuracy. Increasing feature representations can help improve accuracy. Reducing the maximum tree depth may reduce the complexity of the model, but it may also result in some accuracy loss. Reducing the population size can accelerate computation speed, but may affect performance. The adjustment of crossover rate and reproduction rate has a positive effect on improving population diversity and accuracy. A decrease in mutation rate can reduce random searches, but it should be noted that it may lead to a slight decrease in accuracy.

**Fig 10:**
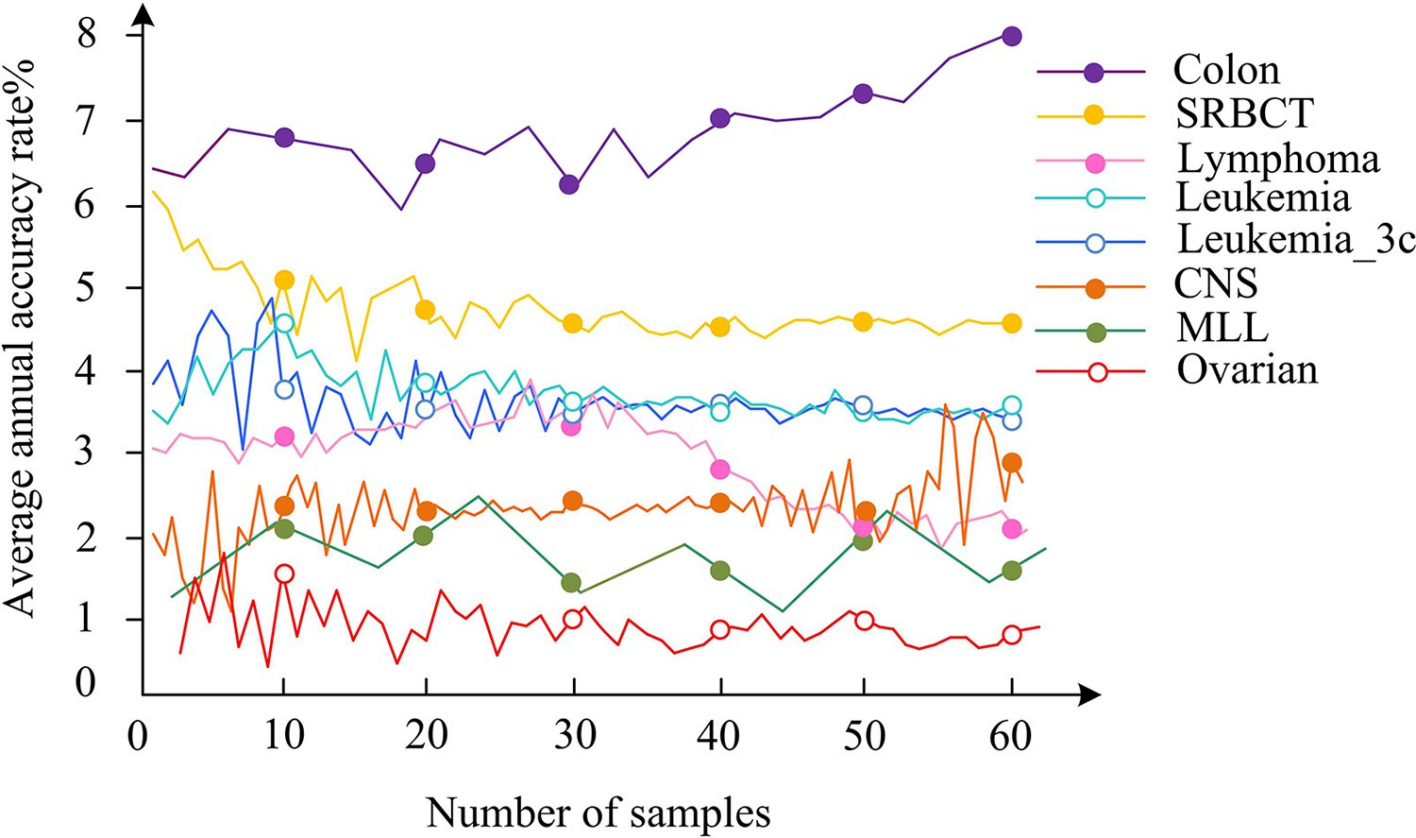
Average accuracy of removing redundant content.

For redundant and unrelated features, the average accuracy of removing redundant content is tested, as shown in Fig 10.

**Fig 11:**
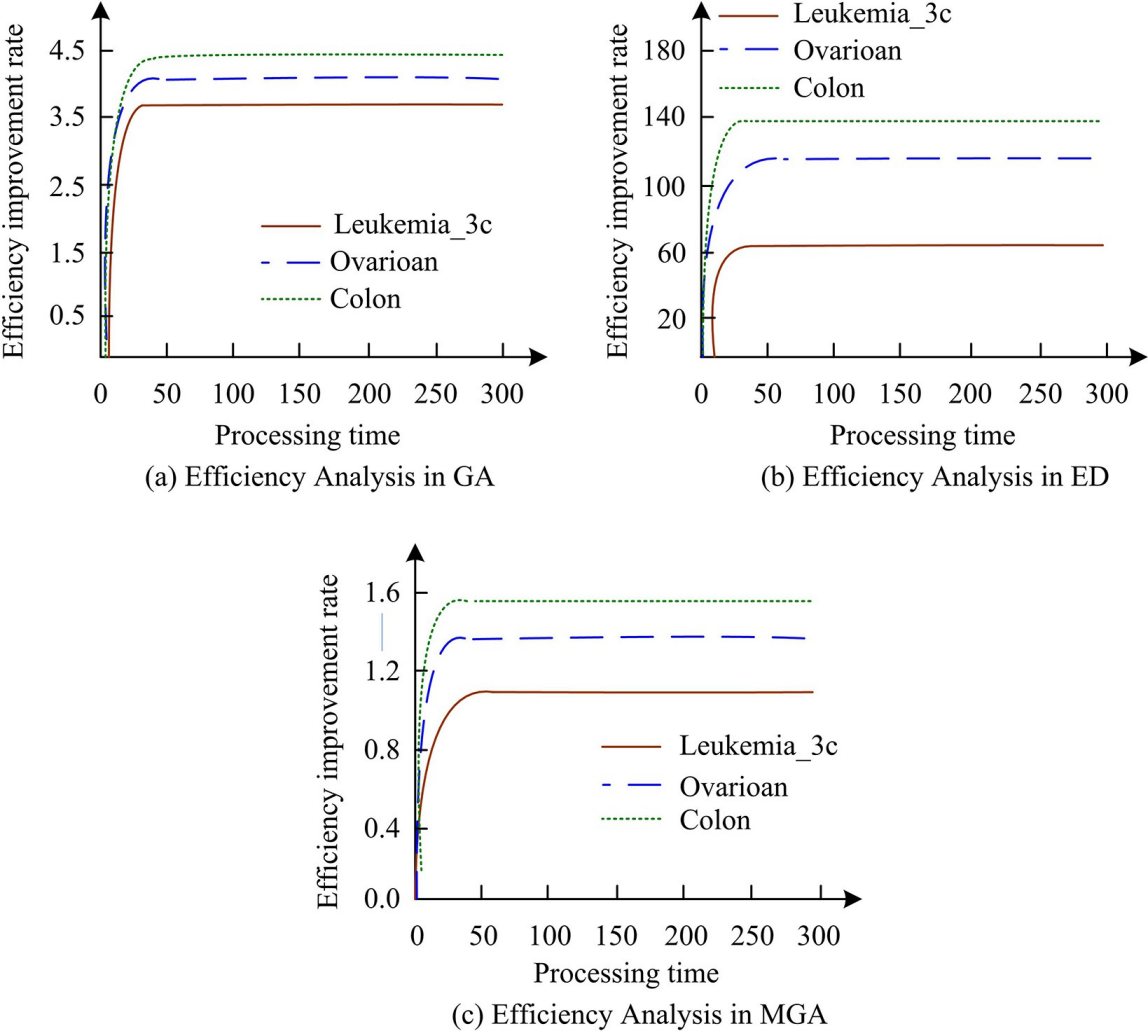
Efficiency analysis of classifier performance improvement in three datasets.

In Fig 10, Colon has the highest average accuracy, followed by SRBCT, Lymphoma, CNS, and Ovarian. The average accuracy of Leukemia and Leukemia_3c is basically the same, but Leukemia_3c is more outstanding. Incorporating specific numeric details, the performance metrics reveal that the peak average accuracy for the Colon dataset is impressive at 95.6%, leading the group. The SRBCT dataset shows a strong performance as well, with an average accuracy of 92.3%. Next is the Lymphoma dataset, accounting for 89.7%. The CNS is 87.4%, and the Ovarian is 85.2%. These figures represent a well-executed methodology in feature selection that significantly enhances the predictive accuracy. For the Leukemia dataset, the accuracy is robust at 88.1%, which is nearly matched by the Leukemia_3c dataset with a slightly higher peak at 88.7%, emphasizing its marginally better performance. There is a small but significant difference in accuracy between leukemia and Leukemia_3c. This indicates that modifications or additional preprocessing steps made to the Leukemia_3c dataset may separate more predictive features, thereby having a positive impact on its classification accuracy. The performance improvement efficiency is compared with the three datasets, namely Colon, Ovarian, and Leukemia_3c, as shown in Fig 11.

In Fig 11, the yellow curve represents Leukemia_3c, the blue dashed line represents Ovarian. The green curve represents Colon. In Fig 11(A), the green curve has the best effect. It is also the most stable, with a time range of 0–300. The green curve is the fastest to reach 4.48, which has been maintaining this efficiency in operation. Compared with the yellow lines, the performance of the blue dashed lines is much worse. In Fig 11(B), the yellow curve has the worst effect, with only less than 60. The green curve and blue dashed line are 140 and 116, respectively. In Fig 11(C), the efficiency of the yellow curve is around 1.05, while the green curve is still as high as 1.34. The classification experimental results of GPFS and LFS methods on eight datasets are illustrated in Table 6.

**Table 6:** Dataset and experimental parameters.

Data set	Method	#F	A-Kn	A-NB	A-C4.5	A-NBT	A-BFT	A-REPT	A-RT	A-RF
Colon	LFS	18.17	78.50	78.85	72.92	78.34	71.85	70.56	70.37	76.65
	GPFS	9.45	75.90	77.42	73.14	72.99	72.96	68.15	68.7	75.97
SRBCT	LFS	65.25	76.88	69.66	62.41	96.36	65.4	40.74	71.11	83.35
	GPFS	19.43	69.83	67.75	64.24	84.06	62.48	41.48	62.41	77.57
Lym	LFS	72.69	82	82	72.62	97.75	78.62	76.67	92.89	96.64
	GPFS	22.35	81	82.65	89.14	97.75	82.29	7,844	90.22	94.54
Leu	LFS	52	90.90	95.35	87.52	94.46	83.3	75.56	84.63	90.95
	GPFS	5.55	88.54	90.95	84.22	92.44	83.7	80.37	87.22	87.95
Leu3c	LFS	48.87	93.14	97.58	82.25	92.69	79.78	76	83.78	92.43
	GPFS	10.3	86.86	92.64	84.43	92.45	80	77.78	83.33	88.62
CNS	LFS	41.15	47.73	57.16	50.46	52.44	52.89	58.44	53.11	49.37
	GPFS	11.26	51.17	51.53	46.87	55.15	49.57	57.12	45.78	45.14
MLL	LFS	70.6	88.11	88.84	67.58	96	66.11	49.63	71.67	86.14
	GPFS	11.9	76	80.37	70.36	78.15	68.23	52.59	66.17	77.98
Ovarian	LFS	31.20	99.51	98.92	95.99	96.76	96.88	95.38	95.69	98.5
	GPFS	3.50	97.94	97.88	95.24	99.2	95.62	94.67	95.64	9,60

In Table 6, among the classification experimental results on eight datasets, except for C4.5, BFTree, and REPTree classifiers, LFS performs better than GPFS on most of the other five classifiers. When the Random Forest is used as a classifier, the classification results on all eight datasets show that the LFS classification performance is higher than the GPFS method. Based on the experimental results, the Colon, SRBCT, Leukemia, Leukemia 3c, CNS, MLL, and Ovarian datasets are more suitable for the LFS method. When using these seven datasets for classification testing, the LFS method has higher classification accuracy than the GPFS method in most cases. On the MLL dataset, when using NBTree classifier for classification, the classification accuracy of the LFS method is 95%, while the GPFS accuracy is only 78.15%. However, the features selected by the LFS method are slightly higher than that of GPFS. In most cases, LFS method is more suitable for feature selection in high-dimensional data. Firstly, to prove the role of the optimized GA in feature selection, ablation experiments are conducted on the initialization process and cross-mutation operation respectively. The test is carried out on high-dimensional data sets. The final results are shown in Table 7.

**Table 7:** Feature selection ablation experiment of optimized genetic algorithm in high-dimensional data processing.

Initial Process	Crossover Mutation	Accuracy	Recall	F1 Score
		147.678	43.010	7.345
Yes		157.242	45.263	7.413
	Yes	47.781	43.033	7.301
Yes	Yes	65.012	48.251	7.445

In Table 7, firstly, the initial process and cross mutation operation is eliminated, verifying the effectiveness of the optimized GA in high-dimensional data processing. Then, the effectiveness of the initial process and cross mutation operation is tested separately. Finally, the synergistic effect of the two is tested. The results clearly show that the initial process alone has no impacts on the results. Some indicators have slightly decreased. The single cross-mutation operation has obvious improvement effect on the original model. The accuracy, recall rate and F1 value are all improved. Compared with the original features, the optimized features are more instructive. When the initial process and cross-mutation operation work together, the results are the best, and the indexes are improved, indicating that the optimized features can achieve better results. This table shows that the initial process and the cross-mutation operations play an important role in the final feature selection result [28].

**Fig 12:**
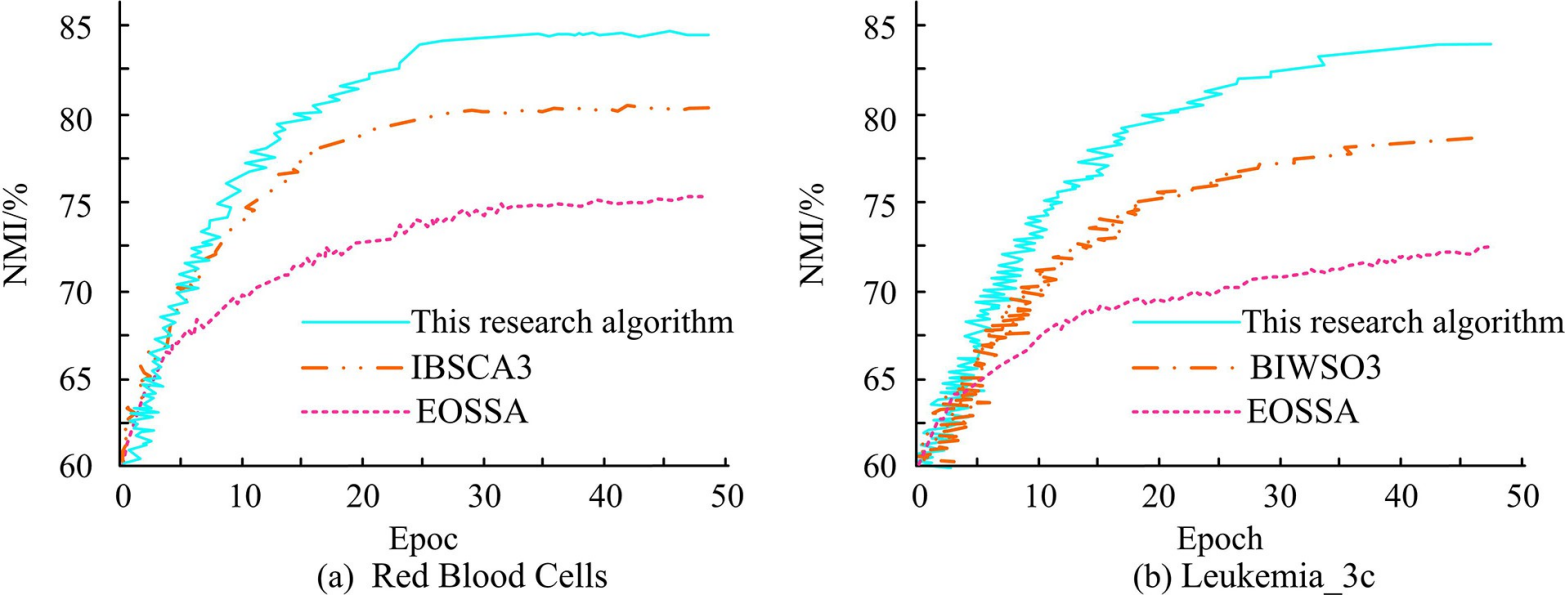
NMI of several genetic optimization algorithms on different datasets.

The normalized mutual information (NMI) of several genetic optimization algorithms on different datasets is shown in Fig 12. In the Red Blood Cells dataset, the NMI of the proposed method is 84.82%, 5.05% higher than that of IBSCA3 and 10.25% higher than that of EOSSA (Improved discrete salp swarm algorithm using exploration and exploitation techniques for feature selection in intrusion detection systems) [29]. In Leukemia_ On the 3c data set, the NMI of the proposed method is 83.35%, which is 6.12% and 11.44% higher than that of BIWSO3 (Binary improved white shark algorithm for intrusion detection systems) [30] and EOSSA (Improved Salp swarm algorithm for solving single-objective continuous optimization problems) [31].

In feature selection for high-dimensional data processing, Chi-square tests and optimization algorithms are used for analysis. Firstly, the study uses Chi-square test to evaluate the correlation between each feature and the target category. The following is the implementation steps. For each feature, a contingency table is created. In this example, the research hypothesis deals with a binary classification problem (e.g. benign and malignant), and the features are binary (e.g. features exist or do not exist). Therefore, the studied contingency table may be as follows.

In Table 8, 105 represents the number of samples with benign features. 95 represents the number of samples with malignant features. 120 represents the number of samples with benign features. 180 represents the number of samples with malignant features.

**Table 8:** Chi-square test analysis.

	Benign	Malignant
Exist	105	95
Absent	120	180

The Chi-square test statistical data for each feature is calculated, as shown in Eq (14).


$$x^{2}=n*{\left(ad-bc\right)}^{2}/\left(a+c)*(b+d\right)*(a+b)*(c+d)$$ (14)

In Eq (14), *n* = *a*+*b*+*c*+*d* is the total number of samples, which is 500 in this example.

After searching the Chi-square distribution table, the freedom degree is 1 (number of categories -1 * number of feature values -1). The corresponding p-value of *x*^2^ is determined. If the p-value is less than the significance level (e.g. 0.05), the study can reject the null hypothesis (feature and category independence) and assume that there is a significant correlation between the feature and category.

After implementing this process, the optimization algorithm is used, such as GA, to find the optimal subset of features that are significantly correlated with the target category. This method combines statistical analysis and optimization search, which can effectively process high-dimensional data and find statistically significant feature subsets, thereby improving the model performance.

## 5. Conclusions and future works

When dealing with high-dimensional data, traditional feature selection methods often has low efficiency and poor accuracy. This study’s optimized GA-based feature selection method has been empirically validated to enhance traditional GA’s efficiency in dealing with high-dimensional datasets. The experimental results underscore the method’s superiority in reducing computational complexity and improving feature subset relevance, which are pivotal in extracting meaningful insights from voluminous data. The fine-tuning of the initialization process, along with the advanced crossover and mutation operations, has shown to significantly refine the search efficacy. The optimization of the fitness function is proven to expedite convergence, facilitating a faster yet more accurate feature selection. The practical implementation of this method has been corroborated through its application to vast, complex datasets, confirming its robustness and reliability in real-world scenarios. High dimensional data contains a large number of features. Only a portion of these features are important for problem-solving. A feature selection solution based on optimized GA is proposed to overcome the "dimension curse" problem encountered in processing high-dimensional data. Various aspects of traditional GAs are improved, including initialization process, crossover and mutation operations, and adaptive functions. According to the experimental results, in the two-dimensional convergence data, GA is the 0 iteration at the optimal value of -0.5. At the optimal value of 3, MGA has 0 iteration. The DE has the optimal value of -1 at 0 iteration. However, after iteration, the optimal values of all three algorithms stabilize at -1.The correct recognition rate of binary encoding has a deviation of 0.0006 before and after. The decimal coding accuracy is 0.0218. In the performance test of ED, the yellow curve has the worst effect, with only less than 60. The green curve and blue dashed line are 140 and 116, respectively. In the MGA efficacy test, the efficacy of the yellow curve is around 1.05, while the green curve remains as high as 1.34. According to the experimental results, on the Red Blood Cells dataset, the NMI of the algorithm in this study is 84.82%, which is 5.05% and 10.25% higher than IBSCA3, EOSSA, and BIWSO3, respectively. This scheme can automatically select the most relevant and representative feature subset from numerous features, achieving the goal of reducing dimensions and improving model accuracy. Good research results have been obtained. However, the domain imbalance corresponding to redundant data is significant, which leads to some shortcomings in dimensions such as running-in and immersion. The optimized GA for feature selection is particularly useful in high-dimensional data analysis but its application is tempered by limitations across various real-world scenarios. The algorithm’s randomness can cause instability in solutions, and its complexity may result in inefficiency, particularly with expansive data sets. In specialized fields such as bioinformatics or finance, where precision and consistency are paramount, these issues could lead to suboptimal performance. Additionally, in areas like image processing or natural language processing, where data volume and dimensionality can be immense, the potential for the GA to converge on local optima due to a poorly designed fitness function can significantly impair the search process and outcome. Despite its adaptability, the algorithm’s limitations necessitate careful adaptation to ensure reliability and efficiency in diverse applications. This is also an area for further improvement in future research. Future research work will focus on further improving the stability and efficiency of algorithms. In particular, research will explore how to more finely adjust the parameters of GA, as well as how to utilize parallel computing and other advanced optimization techniques to shorten computation time. The research also plans to study the fitness function design to more effectively avoid local optima and improve the accuracy and reliability of the feature selection process.

## Supporting information

S1 Data (DOC)
