# Supplementary material for: Feature selection algorithm based on optimized genetic algorithm and the application in high-dimensional data processing
Source: PLoS One. 2024 May 9;19(5):e0303088. doi: 10.1371/journal.pone.0303088 (PMC11081226; doi:10.1371/journal.pone.0303088)
Supplement: S1 Data — (DOC) [file pone.0303088.s001.doc]

Figure 8(a).

| (a) One-dimensional data | | | | |
| --- | --- | --- | --- | --- |
| Iterations | 10^0 | 10^1 | 10^2 | 10^3 |
| Optimal value(GA) | 10^0 | 10^-1.4 | 10^-1.4 | 10^-1.4 |
| Optimal value(DE) | 10^0.3 | 10^0.3 | 10^-1.4 | 10^-1.4 |
| Optimal value(MGA) | - | - | 10^-1.4 | 10^-1.4 |

Figure 8(b).

| (b) Two-dimensional data | | | | |
| --- | --- | --- | --- | --- |
| Iterations | 300 | 600 | 900 | 1200 |
| Optimal value(GA) | 3.00 | 0.01 | 0.01 | 0.01 |
| Optimal value(DE) | 1.52 | 0.01 | 0.01 | 0.01 |
| Optimal value(MGA) | 0.01 | 0.01 | 0.01 | 0.01 |

Figure 8(c).

| (c) 30 dimensional data | | | | |
| --- | --- | --- | --- | --- |
| Iterations | 300 | 600 | 900 | 1200 |
| Optimal value(GA) | 81.21 | 64.35 | 36.84 | 11.32 |
| Optimal value(DE) | 62.12 | 9.23 | 5.31 | 2.32 |
| Optimal value(MGA) | 23.15 | 0.01 | 0.01 | 0.01 |

Figure 9.

| K-value | 1 | 3 | 5 | 7 | 9 | 11 | 13 | 15 | 17 | 19 |
| --- | --- | --- | --- | --- | --- | --- | --- | --- | --- | --- |
| Recognition Rate | 0.9702 | 0.9731 | 0.975 | 0.9716 | 0.9709 | 0.9701 | 0.9698 | 0.9702 | 0.9708 | 0.9709 |

Figure 10.

| Number of samples | 0 | 10 | 20 | 30 | 40 | 50 | 60 |
| --- | --- | --- | --- | --- | --- | --- | --- |
| Average annual accuracy rate%(Colon) | 0 | 1.56 | 0.98 | 1.12 | 1.11 | 1.14 | 1.13 |
| Average annual accuracy rate%(SRBCT) | 6.21 | 5.06 | 4.95 | 4.89 | 4.82 | 4.84 | 4.87 |
| Average annual accuracy rate%(Lymphoma) | 3.12 | 3.21 | 3.32 | 3.14 | 2.87 | 2.12 | 2.04 |
| Average annual accuracy rate%(Leukemia) | 3.57 | 4.61 | 4.02 | 3.97 | 3.94 | 3.98 | 4.07 |
| Average annual accuracy rate%(Leukemia_3c) | 3.89 | 3.95 | 3.97 | 3.94 | 4.02 | 3.98 | 3.91 |
| Average annual accuracy rate%(CNS) | 2.17 | 2.58 | 2.57 | 2.56 | 2.59 | 2.55 | 2.84 |
| Average annual accuracy rate%(MLL) | 1.17 | 2.49 | 2.51 | 2.48 | 2.52 | 2.58 | 2.79 |
| Average annual accuracy rate%(Ovarian) | 0.07 | 1.48 | 1.20 | 1.24 | 1.31 | 1.28 | 1.08 |

**Figure 11**（a）

| (a) Efficiency Analysis in GA | | | | | | | |
| --- | --- | --- | --- | --- | --- | --- | --- |
| Processing time | 0 | 50 | 100 | 150 | 200 | 250 | 300 |
| Efficiency improvement rate(Leukemia_3c) | 0 | 3.51 | 3.52 | 3.51 | 3.50 | 3.51 | 3.52 |
| Efficiency improvement rate(Ovarioan) | 0 | 4.05 | 4.06 | 4.05 | 4.06 | 4.05 | 4.05 |
| Efficiency improvement rate(Colon) | 0 | 4.48 | 4.49 | 4.49 | 4.49 | 4.49 | 4.49 |

Figure 11（b）

| (b)Efficiency Analysis in ED | | | | | | | |
| --- | --- | --- | --- | --- | --- | --- | --- |
| Processing time | 0 | 50 | 100 | 150 | 200 | 250 | 300 |
| Efficiency improvement rate(Leukemia_3c) | 0 | 61.01 | 61.02 | 61.01 | 61.02 | 61.01 | 61.01 |
| Efficiency improvement rate(Ovarioan) | 0 | 128.24 | 128.24 | 128.25 | 128.24 | 128.23 | 128.24 |
| Efficiency improvement rate(Colon) | 0 | 139.28 | 139.30 | 139.28 | 139.31 | 139.29 | 139.30 |

Figure 11（c）

| (c) Efficiency Analysis in MGA | | | | | | | |
| --- | --- | --- | --- | --- | --- | --- | --- |
| Processing time | 0 | 50 | 100 | 150 | 200 | 250 | 300 |
| Efficiency improvement rate(Leukemia_3c) | 0 | 1.09 | 1.11 | 1.09 | 1.11 | 1.09 | 1.11 |
| Efficiency improvement rate(Ovarioan) | 0 | 1.40 | 1.39 | 1.40 | 1.39 | 1.40 | 1.39 |
| Efficiency improvement rate(Colon) | 0 | 1.59 | 1.58 | 1.59 | 1.58 | 1.58 | 1.59 |

**Figure**12.(a)

| Epoc | 0 | 10 | 20 | 30 | 40 | 50 |
| --- | --- | --- | --- | --- | --- | --- |
| NMI/%(This research algorithm) | 60.00 | 71.89 | 81.95 | 84.37 | 84.39 | 84.46 |
| NMI/%(IBSCA3) | 60.00 | 72.5 | 79.65 | 80.14 | 80.16 | 80.18 |
| NMI/%(EOSSA) | 60.00 | 68.01 | 75.02 | 75.54 | 75.56 | 75.58 |

**Figure**12.(b)

| Epoc | 0 | 10 | 20 | 30 | 40 | 50 |
| --- | --- | --- | --- | --- | --- | --- |
| NMI/%(This research algorithm) | 60.00 | 71.51 | 81.57 | 83.24 | 84.01 | 84.04 |
| NMI/%(IBSCA3) | 60.00 | 72.54 | 79.65 | 80.52 | 80.56 | 80.57 |
| NMI/%(EOSSA) | 60.00 | 68.24 | 75.45 | 75.57 | 75.61 | 75.62 |
